# Racial variations in booking haemoglobin of primigravidae in Malaysia: a prospective study

**DOI:** 10.1186/1756-0500-6-173

**Published:** 2013-05-01

**Authors:** Albert Chao Chiet Tan, Eugene Weng Kong Leong, Ai Chen Chua, Foong Ming Moy

**Affiliations:** 1 Department of Obstetrics and Gynaecology, University Malaya Medical Centre, Kuala Lumpur, Malaysia; 2 Department of Social and Preventive Medicine, University Malaya Medical Centre, Kuala Lumpur, Malaysia

**Keywords:** Haemoglobin, Primigravida, Pregnancy, Race, Anaemia

## Abstract

**Background:**

Variations in racial haemoglobin had been previously described in multiple studies locally and abroad. This study was conducted to quantify the differences in haemoglobin of booking primigravidae amongst the three major races in Malaysia at the antenatal clinic of University Malaya Medical Centre, Kuala Lumpur.

**Findings:**

One year prospective study of booking full blood count sample of primigravidae taken in one centre was conducted. Multiple comparative analyses of the booking haemoglobin were performed using the One-way ANOVA comparative mean test in each trimester. 622 primigravidae without any known history of haematological disorders were recruited into the study. The mean haemoglobin for the Indian race was the lowest compared to the two other races in the second and the third trimesters, and it was found to be statistically significant lower (p- value 0.001) than the Malay race in the second trimester. It was also found that the Indian race had a significantly higher incidence of moderate to severe anaemia (p- value: 0.029). The prevalence of anaemia in our study population is also significantly higher in the Indian population (p- value: 0.01).

**Conclusions:**

The findings from this study have established that there is racial preponderance to anaemia in pregnancy. The Indian race is at a higher risk of having anaemia in pregnancy particularly in the second trimester.

## Findings

### Background

Malaysia was a previous crown colony of the British Empire until independence in 1957. As a result of being previously colonized by European settlers, Malaysia has a multiracial society which comprises of citizens who are direct descendants from China, and India; both forming a significant minority in Malaysia. Since independence, Malaysia had undergone several economic transformation programs which had favoured certain ethnic groups in order to reduce the disparities amongst the races in terms of economy, education and healthcare distribution [1]. Recent studies had suggested that there were still discrepancies in maternal healthcare provision amongst the races [2,3]. This paper was conceptualized to quantify the differences in maternal haemoglobin amongst the three major races in Malaysia, and we sought to use the booking haemoglobin of primigravidae as a surrogate marker for this purpose.

Anaemia in pregnancy has been associated with the delivery of low birth weight infants, pre-term birth, and maternal mortality [4-7]. The prevalence of anaemia is higher in teenage group, non-urban areas, more prevalent in lower socioeconomic status groups, and in higher parity (five or more pregnancies) [1,9-12]. Race as an independent risk factor for anaemia had been described in multiple studies [2,4,12,13]. In addition, race- specific criteria in screening for iron deficiency anaemia had once been considered [8]. There have been several studies done to describe the distribution of anaemia in Malaysia [2,11,13,14] but, none of these studies had focused on the well-being of primigravidae alone.

In a recent Malaysian cross-sectional survey study done by Haniff J et al., [2] from their multicentre analysis of all maternal booking venous and capillary blood, they found that there were significant associations of anaemia with the ethnic group and the gestational age [2]. In their study, the average haemoglobin level of the Chinese in Malaysia was found to be significantly higher than the haemoglobin level of the Malays [2].

Therefore, there is a need to clearly establish the above differences with a prospective study of booking mothers in a controlled setting. This study aims to provide a qualitative and quantitative analysis of the differences in booking haemoglobin for primigravidae amongst the Malays, Chinese and Indian population from an urban obstetrics centre with respect to the mean haemoglobin, severity and prevalence of anaemia.

## Materials and methods

This is a prospective study of primigravidae booked at the Antenatal Clinic of University Malaya Medical Centre, a tertiary hospital located in Kuala Lumpur, Malaysia. It serves as a referral centre and an urban obstetrics centre for a population of about eight million. This population consists of 45.9% Malays, 43.2% Chinese, 10.3% Indians and 0.6% other ethnic groups which consist of Eurasians, and other indigenous races from East Malaysia [15]. Race was determined by the name and racial identification documents on the marriage certificate.

Primigravidae booked in UMMC antenatal clinic from February 2010 to January 2011 were recruited into the study. Data regarding racial identification, date of birth, gestational age, and previous medical conditions were collected. Their antenatal booking full blood count (FBC) samples were then taken. All primigravidae found to be anaemic were then screened for thalassaemia, and other common haematological conditions. Primigravidae who had been diagnosed with sickle cell trait/disease, thalassemia trait/disease, or any myelodysplastic disorders were excluded from the study. Booking primigravidae from foreign countries were also excluded from the study. University Malaya Medical Centre Ethics Committee approved the proposal for this study and consent was obtained from all booking primigravidae.

A total of 3- 4mL of venous blood sample were taken from the antecubital vein of each woman by venepuncture. The sample was then put into a dipotassium EDTA specimen for analysis using a spectrophotometer (Sysmex XE 5000, Japan).

The data were then analysed with the One-way ANOVA comparative mean test separately for the first, second and third trimesters. The statistical package SPSS version 17 was used for the One-way ANOVA data analysis and Microsoft Excel 2010 was used for the Chi- square test. The level of significance was set at p-value ≤0.05. Subsequently, a post-hoc Bonferroni correction method of analysis with their respective level of significance was calculated for each race.

## Results

There have been several studies and poster presentations done to assess the level of haemoglobin for pregnant ladies in Malaysia; however none had focused on the booking well- being of primigravidae and was done in a prospective setting. Therefore, the findings from this study are important particularly in the management and public health allocation for women in their first pregnancy.

622 primigravidae without any known history of haematological disorders were recruited into the study, 384 (61.7%) were Malays, 105 (16.9%) were Chinese, 124 (19.9%) were Indians, and 9 (1.5%) were from the other minority races of Malaysia. A total of eight primigravidae booked were excluded from the study as they were diagnosed with thalassaemia. During our study period, we did not encounter any primigravida that was a result of an inter-racial marriage. Our study population were reported to be clinically well during the venepuncture, they were not actively bleeding from any discernible source. There were 33 primigravidae whose data were not available and incomplete, and were therefore excluded from the study.

The majority of the primigravidae were booked in the second trimester (51.8%). There were 45 primigravidae booked in the first trimester- 34 Malays, 4 Chinese, 6 Indians and 1 other; 322 booked in the second trimester- 197 Malays, 55 Chinese, 64 Indians and 6 others; and 255 booked in the third trimester- 153 Malays, 46 Chinese, 54 Indians, and 2 others.

From our analysis of the data collected, we found no statistically significant differences in the booking haemoglobin of primigravidae in the first trimester and third trimester.

However, we did find that there were statistically significant differences in the booking haemoglobin of the Indians and the Malays, p- value 0.001 in the second trimester. The booking haemoglobin and their respective 95% CI for each trimester are summarized in Table 1.

**Table 1:** The mean haemoglobin with their respective 95% CI in each trimester for the three major races

	**Haemoglobin (g/dL)**		
	**1**^**st **^**Trimester**	**2**^**nd **^**Trimester**	**3**^**rd **^**Trimester**
Malay	12.58±0.30	11.85±0.14	11.90±0.19
Chinese	12.83±1.82	11.79±0.34	12.21±0.40
Indian	12.73±1.30	11.29±0.29	11.78±0.39

**Figure 1:**
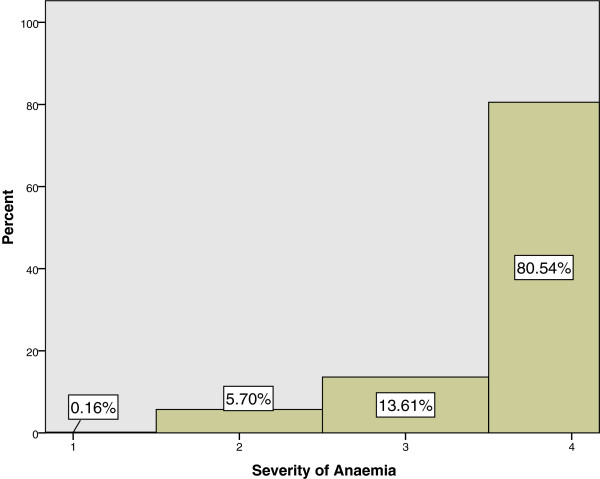
**Prevalence of anaemia in primigravidae with their respective severity.** (Mild anaemia: 10.0-10.9 g/dL, moderate anaemia: 7.0-10.0 g/dL, and severe anaemia: less than 7.0 g/dL).

**Figure 2:**
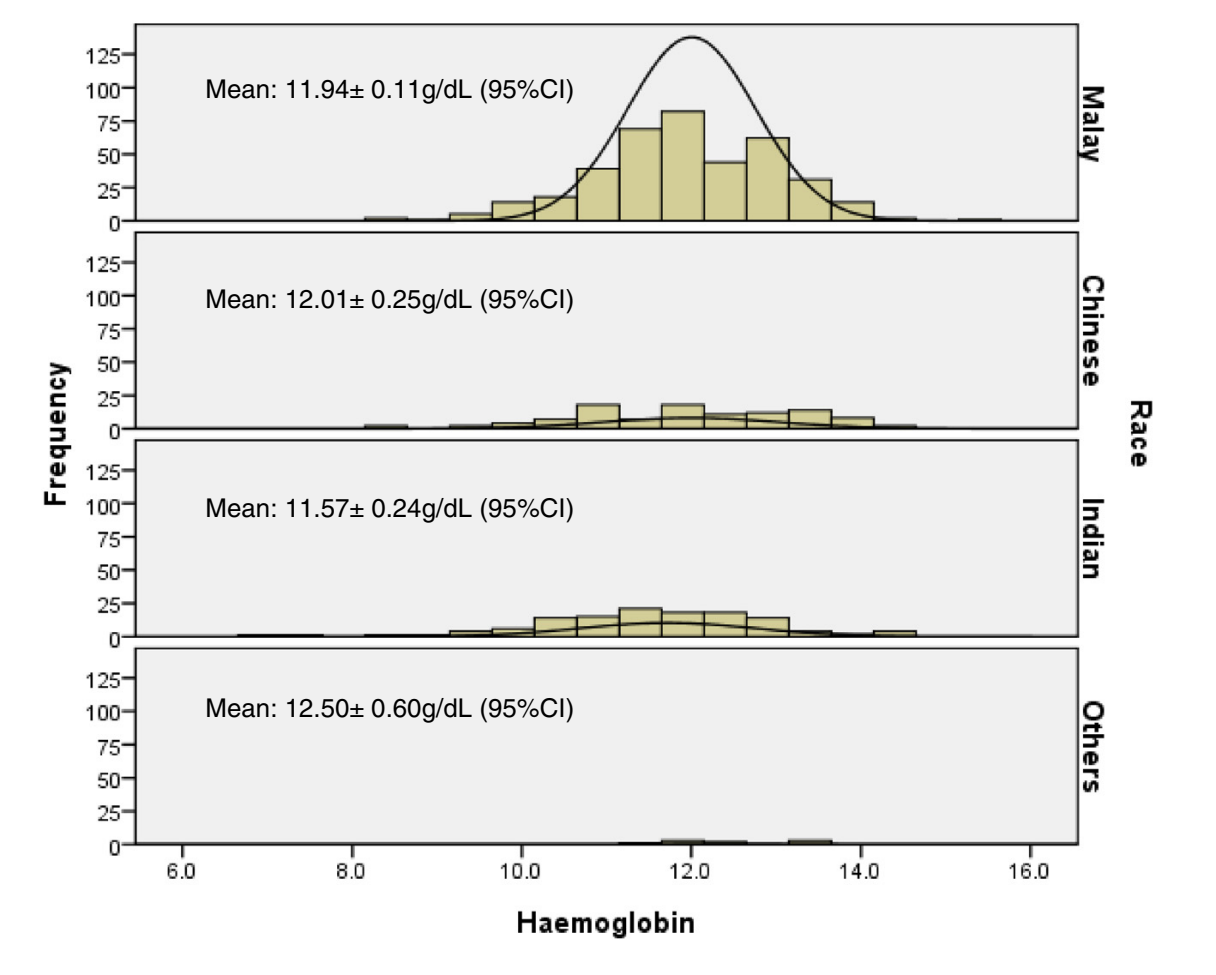
**The distribution of haemoglobin levels amongst the races in Malaysia.** Frequency: number of primigravidae; Haemoglobin: Mean±95% CI g/dL.

Figure 1 shows the distribution of anaemia with their respective severity for all the trimesters, and Figure 2 shows the distribution of the haemoglobin levels in each race for all the trimesters.

## Discussion

### Age at first booking

The study population consisted of mainly mothers between the ages 20 to 30 years old. There were only 0.3% of booking mothers below 18 years of age, 84.4% of the study population were from 18 to 30 years old, 15.0% were from 31 to 40 years old, and 0.3% of the study population were above 40 years old. The racial demographic distribution in this study population of 61.7% Malay, 16.9% Chinese, 19.9% Indians and 1.5% others closely reflects the expected racial distribution of the Malaysian population [15].

The mean age for the participating primigravidae is 27.58 years (SD3.82). The average age for the Chinese primigravidae is 29.20 years (SD 4.79) whilst, for the Malay is 26.78 years (SD 3.21) and the Indians is 28.70 years (SD 3.99).

**Figure 3:**
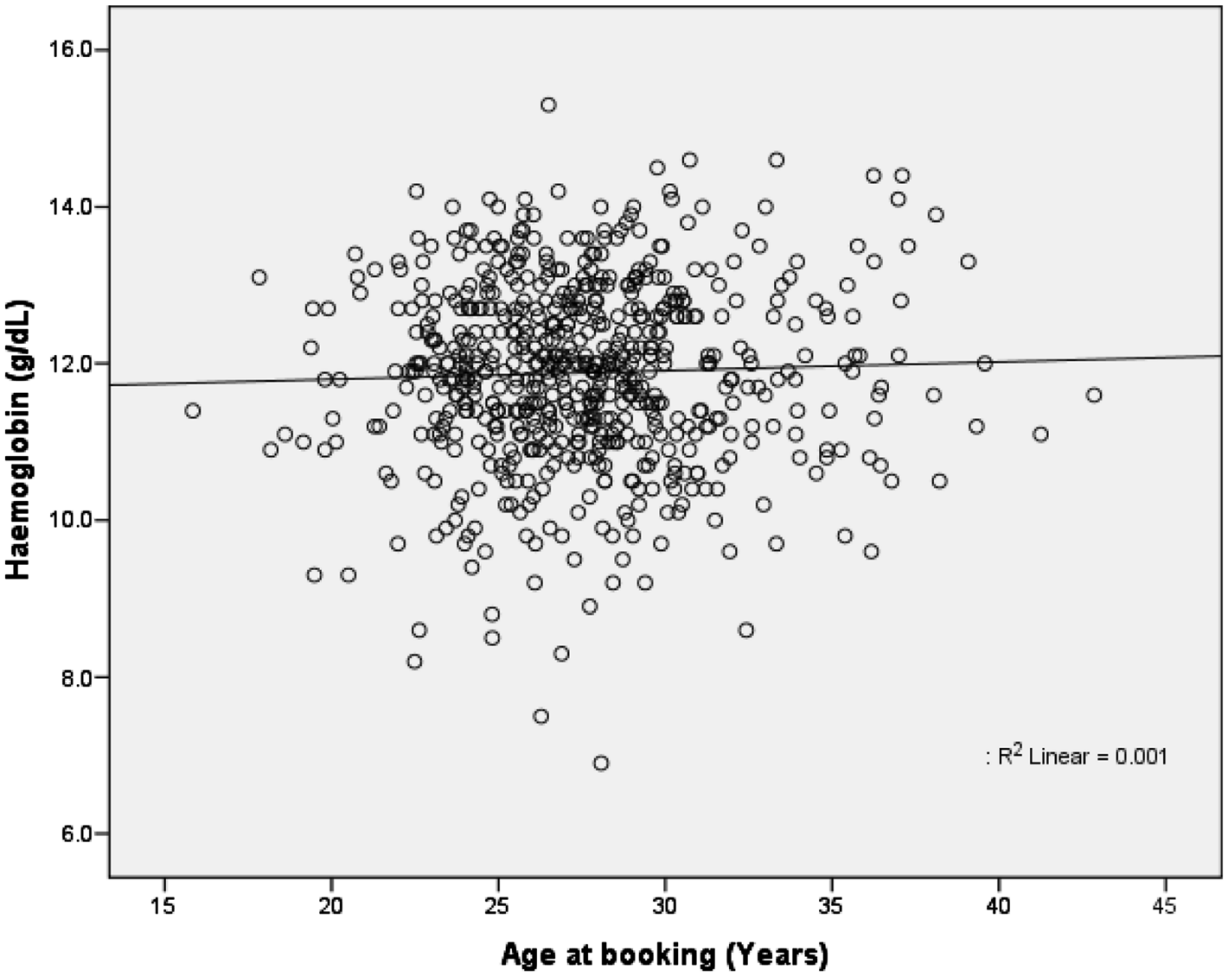
**The distribution of haemoglobin (g/dL) in relation to the age of the primigravida (years).** P- value 0.055.

We also examined for any possible correlation between the age at first booking and the level of haemoglobin, and from our analysis it was found that there was no statistically significant correlation (Pearson’s correlation 0.035, 2-tailed significance of 0.378). Figure 3 shows the Scatter plot for this analysis. Therefore, regardless of the age at booking, the level of haemoglobin would be comparably homogenous.

### Prevalence of anaemia in primigravidae

Anaemia was present in 120 (19.3%) primigravidae of our study population, it is relatively low compared to the WHO estimate of 51% for developing countries [16]. This level is comparable to the levels found in developed countries, 5.0-19.9% [16].

62 (16.1%) of the Malay population were anaemic whilst, 23 (21.9%) of the Chinese population and 35 (28.2%) of the Indian population were anaemic. There were statistically significant differences amongst these three races with regards to the prevalence of anaemia from our Chi- square analysis (p- value 0.01); the Indian race was found to have the highest prevalence of anaemia.

Our findings were different from the study done by Haniff J et al. [2], and this could have been due to the differences in our study design and inclusion criteria. Our study compared only mothers in their first pregnancy, and it is therefore not affected by higher parity haemoglobin status which is found more commonly in Malay mothers for this region [17].

### Severity of anaemia in pregnancy

During our study period, the lowest recorded haemoglobin level was found in the Indian population, having a haemoglobin value of 6.9 g/dL. This was later diagnosed to be due to iron deficiency anaemia. Figure 1 and Table 2 further describes the distribution for the severity in anaemia amongst the three races. There were no other primigravidae from the other races found to have severe anaemia during our study period; only moderate and mild anaemias were found in other races.

**Table 2:** The number of primigravidae with their respective severity of anaemia amongst the three major races in Malaysia

	**Severe-to-moderate anaemia**	**Mild anaemia**	**Non- anaemic**
Malay	16 (4.2%)	46 (12.0%)	322 (83.8%)
Chinese	7 (6.7%)	16 (15.2%)	82 (78.1%)
Indian	13 (10.5%)	22 (17.7%)	89 (71.8%)

The prevalence for the severity of anaemia is represented in percentages for each respective race.

A Chi- square analysis between the three major races (Malay, Chinese and Indian) with their respective severity of the anaemia (severe-to-moderate anaemias, mild anaemia and non- anaemic) showed that there were statistically significant differences between the distribution of severity and the races (p- value 0.029). The Indian race had the highest tendency of developing moderate to severe anaemias when compared with the other two races.

### Limitation

The other possible causes that can affect the haemoglobin such as the tea intake, vegetarians, smoking status, worm infestation, vitamin intake, and maternal body mass index were not corrected for during the data collection.

## Conclusions

The Indian race has been shown to have a higher risk for anaemia in terms of its severity and prevalence when compared with the other two races. This study has thus shown that there was significant disparity in health care distribution and that there was racial preponderance to anaemia in pregnancy in our study population. Therefore, appropriate measures need to be taken to reduce the incidence of anaemia in pregnancy for the Indian race in order to avoid adverse pregnancy outcomes related to anaemia.

## Abbreviations

CI: Confidence Interval; FBC: full blood count; Hb: Haemoglobin; UMMC: University Malaya Medical Centre.

## Competing interests

The authors declare that they have no competing interests.

## Authors’ contributions

ACCT designed the study, was involved in the data collection, analysis of data, in the writing of the manuscript, and is the lead investigator. EWKL was involved in the design of the study, data collection and was involved in the writing of the manuscript. FMM was involved in the analysis of data and the writing of the manuscript. ACC was involved in the data collection and the writing of the manuscript. All authors read and approved the final manuscript.
